# A Study on the Correlation between Intelligence and Body Schema in Children Who Practice Chess at School

**DOI:** 10.3390/children9040477

**Published:** 2022-03-30

**Authors:** Vlad Ionuț Stegariu, Beatrice Aurelia Abalasei, Marius Stoica

**Affiliations:** 1 Faculty of Physical Education and Sports, Alexandru Ioan Cuza University of Iaşi, 700506 Iasi, Romania; beatrice.abalasei@uaic.ro; 2 Center for Training and Professional Development, National University of Physical Education and Sports, 060057 Bucharest, Romania; mariusstoica08@gmail.com

**Keywords:** psychomotor intervention, childhood, primary school childhood, psychomotor development

## Abstract

The role of intelligence in chess is crucial because the game involves a situation of adversity between two players whose goal is to checkmate the opponent’s king. Due to the complex nature of the game and the huge amount of information needed to become a professional chess player, the ability to receive, analyze, sort and use abstract notions is essential. A total of 67 children from the third grade were selected and tested twice, initially and finally, to establish the level of body schema and intelligence. The Raven test was used to numerically quantify their intelligence and the Goodenough test was conducted for the body schema. We used the paired samples T-test to highlight the statistical difference between the results and performed a simple linear regression to see if the level of intelligence is a predictor of the body schema. There is a linear relationship between intelligence and body schema, and we can use the first one to predict the evolution of the second. In conclusion, body schema can be educated through chess lessons, and this will lead to better psychomotor development.

## 1. Introduction

The chess activity has a deep character in which the ideas of the two players are guided by strategic principles and tactical procedures to reach the final goal—checkmate. The different ways of moving the pieces force the players to use logic-based creativity. The queen, the bishop and the rook move linearly, the knight, the king and pawns have different trajectories combined with special rules. The synchronicity of the adversarial nature of the game with the possibility to end the game in a draw offers the opportunity to involve a large number of different types of characters in the world of chess.

In order to better understand the depth of chess, one must know the characteristics of its foundation. Any field based on a significant amount of theoretical knowledge requires many hours of study that can only be obtained if the player has the ambition or increased passion. Campitelli [1] and Gobet [2] addressed the deliberate practice issue, and the main conclusion was that it is necessary, but not sufficient. As in any other sport, the amount of training hours required for performance is huge, and this is explained by the fact that the grandmaster knows over 100,000 specific patterns [3]. Also, deliberate practice, which is mandatory but not sufficient [4,5,6,7,8,9], has a minimum threshold of 3000 h [1], which is needed in order to become a grandmaster. These classes do not involve systematized and coordinated training by a coach, such as training games. Chess requires more cognitive skills and sophisticated problem-solving abilities [10,11,12], thus providing a good opportunity to study the mechanisms underlying cognitive expertise. Studies have shown that the frontal and posterior parietal areas, which are known to be involved in the orientation of attention, perception and working memory, are engaged in the game of chess [13]. Systematical chess practice develops several important skills in solving mathematical problems [14,15,16], such as maintaining a high level of attention [17] and focusing on tasks [18], perseverance in pursuing goals, creativity [19], recognizing strategic information in situations and using it in planning strategies, critical reflection on one’s actions and predicting the course of events [20]. There is a statistically significant correlation between intelligence and chess performance [21,22,23,24,25,26,27], thus the child who excels at a certain school subject has the chance to achieve better performance in chess.

During a game, there will be several complicated situations, called critical moments, in which the decision made must be logical. By practicing chess, these moments that tend to create panic are improved [28], and their management becomes more successful with time. Also, in the case of professional players, it was found that key moments are identified faster, and their approach is based on analytical logic [29].

The similarities between the evolution of the child and that of the chess player are multiple and in the case of the formation of representations the situation is identical. At first, their meaning is wrong or incomplete, but the study of standard positions, the discovery of tactical motifs and strategic principles and deliberate practice leads to the formation of complex general patterns. Observing and studying chess games played under strong time pressure revealed that the way chess players think is not affected by this [29,30]. Also, the efficiency of thinking is correlated with the number of complex patterns that the chess player knows, and the speed with which they are updated makes the difference between a good player and a top one [18].

After presenting the aspects of the game of chess and how they influence the intellectual development of children, in the following paragraphs, we will analyze the evolution of the body schema and its connection with the game of chess.

The beginning of school life involves some major changes in the body schema because the characteristics of the new environment differ in an overwhelming proportion from the one inside the family. During this period, there is a thirst for knowledge which, due to differences in perception, will generate conflicts, minor but repeated, with the parents [31]. Because one’s own body schema is not synchronized with the real one, personal opinions do not correspond to those of the teacher, thus triggering a mechanism of involuntary reassessment of knowledge already accumulated. In the context of a school failure, the child tends to lie in an attempt to justify the parents’ expectations [32]. In the game of chess, the rules are clear and accepted by both players before the game starts. Here, lies and hypocrisy are punished immediately. The chess body schema is constantly changing since the first tournament, resulting in an acceptance of unexpected adjustments. The child is subjected to a continuous adaptation mechanism, and these retouches will outline the shape of the body schema as faithful as possible to reality. Due to the system of measuring chess value, the landmarks are clear, and the effects of a victory or defeat spread to the perception of oneself. In the game of chess, the development of this psychomotor behavior is achieved simultaneously with the use of the game clock. It is often utilized in training in order to simulate decision-making situations under time pressure. The main consequence is the creation and development of neural connections [33] designed to shorten the time of information by processing and providing a prompter response [34,35].

School activities involve multiple changes in the child’s daily life in which he must coordinate the movements of his own body, locate himself in relation to objects or colleagues, order the objects and actions that follow each other, appreciate them and appreciate himself in relation to others, and so the perception about his own body continuously evolves.

The interaction of the individual with the environment or its constituent elements is a continuous process that has as its main goal the formation of the body schema. The representation of the body schema undergoes changes, sometimes regressive, depending on the sensory inputs [36]. Maravita [37] believes that, from a neurosensory approach, the body schema is based almost entirely on information received visually and tactilely. They produce internal representations [38] that have the role of generating a response at the cerebral level, which means there is a close connection between intellectual ability and the level of the body schema. In order to be able to understand the body, the brain needs an effective and accurate integration of the information received—this way, the representations become coherent [39]. The attribution of meaning is achieved only when the individual is aware of the way in which elements of the environment influence their body’s evolution. Therefore, the need to develop a thought process is essential during primary school. The child is forced to live in several social environments that have different peculiarities, and the transition is sudden and definitive, for example, from kindergarten to school. The preschool period is shaped by fantastic elements that influence the child’s perception, both of the environment and himself. Cognitive limits restrict the possibilities of testing, which means that, in order to observe the level of the body schema, an open test must be chosen. Liu [39] proposes the example of a prism to explain the role of cognitive processes in the formation of the body schema, where the proportions, color and shape of objects differ from the real ones. In children, given that their motor experience is limited, the body schema takes the shape of what they know, not what they see. If we examine the drawings of a child, we can observe that the content is related to their mental development [40] and as they grow, the drawings become intelligible due to the fact that they are able to insert fine details [41] of which they were previously not aware.

According to Raimo [42], at the end of primary school, pupils manage to outline structural representations similar to that of the adult. This upward trend is continued until the age of 60, at which point the perception of self regresses [43]. It should be noted that by the age of 10, the elements of the face were better defined than the others [44], and one possible explanation would be that the main stimuli used to cumulate information from the environment is located at the level of the head. According to Kagerer [45], general dynamic coordination in children shows a variability inversely proportional to age. The 7-year-old child and the 10-year-old will be able to fit the elements of the face in a circle that represents the individual’s face, but the difference is made in terms of positioning and proportions. This justifies the fact that the Goodenough test provides a clear picture of the evolution of the body schema relative to the time axis.

The perception of one’s own body is essential in everyday activities and contributes to the development of self-consciousness [37]. The localization of body segments, even in children, is carried out involuntarily, so, we can talk about control of them in relation to spatiality. The complexity of the described process and its transformation into automatism gives us clues as to how the body schema evolves (or strengthens). In the case of the child who practices chess, due to the clearly defined hierarchies [46], any result generates a response at the cerebral level. If we take the case of chess progress, then we can speak of a repetition of favorable results against a category of players, so, the evolution of those games will follow a pattern. This process results in a substantial change in the body schema, especially in the way the child perceives his own image in relation to the competition. Medina [47] and Schowebel [48] talk about these changes and confirm the existence of a body schema in continuous evolution. There are an increased number of internal systems that help us shape our own three-dimensional images according to the environments in which we live.

Spatial-temporal orientation is a psychomotor conduct that is closely related to the development of the body schema. It is easy to understand the role in the maturation process of children. Integration into the environment, which is essential for the formation of the body schema, is not possible without a good definition of spatial and temporal landmarks, and this process takes shape only during puberty. To give a clarifying example, we can observe team sports, for example, football or handball, where up to the age of 13–14, there is no emphasis on collective tactics, but on individual technique. 

Given that this research is focused on observing a state of affairs, that of teaching chess in school as an optional subject, where there was no selection bias, choosing the design of research and the tests was the most important step. Observing and analyzing the opinions presented above, we concluded that the body schema is the projection of the three-dimensional image of oneself. Its formation is the result of our motor actions from day one.

Due to the similarities between the evolution of the body schema and a chess game, we assume that through chess we can improve the level of the body schema. 

## 2. Materials and Methods

The purpose of the research is to observe if one hour of chess, implemented in the school curriculum, influences the intelligence and the level of the body schema. Based on this, we used a simple linear regression to observe if the level of intelligence can predict the level of the body schema. Because the research design includes two tests, initial and final, we highlighted the statistical differences with a paired samples *t*-test. Cohen’s d was used for checking the probability of superiority between the initial and final tests. It is used as a complement to the *t*-test or ANOVA to show numerically how big the difference is. 

We tested 67 children from the third grade who study chess in school once a week during their normal schedule. The activity was included in the school curriculum as an optional subject. At this school, parents, by voting, decide which optional subject children will study the next year. In the case of the two classes that, combined, make up the entire group of subjects, the discipline of Physical Education and Sport—chess was chosen to start with the first school year. The decision was maintained without interruption until the end of the primary cycle. Students were initially tested in the last week of the first semester of the third grade, and the final test took place at the end of the first semester of the fourth grade. We mention that no student has any special condition, neither of a cognitive nor behavioral nature. Even so, their academic path falls within normal limits without any external intervention. We chose these two classes because they had the same path—meaning that they started studying chess in the first grade (First Class) and with the same teacher. Now, they are going to finish the primary school cycle and move on to different schools. There was no selection bias, meaning that no child was disqualified and because of this, we did not carry out a power analysis to determine if it was a correct sample size. We wanted to see the results at the end of the primary school cycle.

We did not choose a control group because the children’s evolution was related to the age test scales. For this reason, testing was carried out at the end of semester 1 of the third and fourth grades. By choosing this period, we were able to follow their evolution over the course of a calendar year, not a school year (which lasts less than nine months). The purpose of the test was to quantify their evolution and not to compare the effects of practicing chess with those of studying another optional (for example: linguistic (third language) or health education).

According to Sala [16], experimental design with a control group is not effective. To prevent the placebo effect, a third group is needed that has the status of the one who practices chess. The students were tested at the school in the presence of their educator, and the activity was perceived as any other test during the year. Parents were aware of these tests since the first school year.

The group was composed of 31 boys and 36 girls. They started studying chess in the first grade and had the same teacher whose main qualification was physical education—chess. 

The children were subjected to two assessments that aimed to determine their intelligence and body schema. Both skills are extremely important in the educational process, defining the character of the adult over the years. We used the Raven progressive matrices and the Goodenough test. Raven progressive matrices are the most popular nonverbal test aimed at quantifying intelligence. Solving the tasks involves discovering a rule for the development of the elements. Certain mental functions are correlated with general intelligence, so, by solving nonverbal tasks that activate those mental functions, we can determine the I.Q. coefficient. It also examines the ability to identify a distinct element from a group, the spirit of observation, the ability to operate with abstract notions and also the short-term memory.

The Raven test comprises 60 items divided into 5 series of 12. Each item presents an abstract drawing with a missing part, and the child must choose the missing piece from a group of 6, in the case of series A, B, C or 8, in the case of series D and E. Each series addresses a different topic, namely:

- Series A—establishing relationships within the matrix;
- Series B—analogies between pairs of figures;
- Series C—progression of matrix elements;
- Series D—permutations of the elements within the matrix;
- Series E—decomposition of matrix elements [49].

The final result is composed of two factors, the number of correct answers out of the total of 60 items and the age of the student. For each age group, a correct number of responses corresponds to a percentile rank. Its value provides the child’s level of intelligence. We recommend consulting the following works to better understand how this test can be applied, Małkiński [50], Qiu [51] and Waschl [52].

The Goodenough test is used to investigate personality, but this test can also reveal the level of intelligence. The test involves the drawing of a man on a piece of paper without giving any other directions. The child performs the task as he considers, and when he has difficulty or wants some explanations, the examiner offers an impassive answer: “Do as you want” [53].

The score is punctual for each element present in the child’s drawing, and the maximum score is 50 points. These elements are classified into 18 groups, having one thing in common. The 18 groups are: presence of head, presence of legs, presence of arms, presence of torso, tangency of limbs, presence of neck, face elements, presence of hair, presence of clothes, presence of fingers, presence of joints, body proportions, presence of heel, motor coordination, ears, the presence of the chin and forehead, eye details and silhouette. The obtained results reveal the level of development of the body schema, but also the I.Q. related to it.

The Goodenough test has a couple of fundamental similarities with chess. The conduct of a chess game is guided by the principle of causality, in the sense that each move provokes a response from the opponent. The evolution of the game involves a continuous adaptation and recalculation of one’s own forces in relation to the environment. Each piece, similar to each part of the body, can lead to the achievement of clearly defined goals. Success or failure to achieve goals forces the child to observe, analyze and synthesize the new position. This repetitive process is identical to that of the formation of the body schema, the main difference being that in the first example the child performs moves, and in the second, motor actions. Novice chess players will repetitively move a couple of pieces, neglecting some of them, and the effects are immediate. This awareness of all forces leads to the conception of intelligent plans, but in order to reach this stage, the child must understand the role of each piece. 

Also, the chessboard proposes the development of spatiality both at the superficial level (the piece inside the square) and also at a deeper level (placing the pieces on the most powerful squares). Neglecting certain parts of the body in the Goodenough sample suggests that the level of the body schema is deficient, and the perception of oneself needs improvement.

The Goodenough test was also chosen because it is suitable for the age of the subjects. The results are important considering that they aim to validate their chess progress, level of intelligence and academic path.

Both tests do not include a time limit but, in general, the first test takes around 25–30 min and the second one, up to 10 min. The test coordinator is not allowed to help or influence the children at all, not even through gestures or posture. 

We used IBM SPSS Statistics 20 software to generate the descriptive statistics and to determine the *p*-value of the paired samples *t*-test and Linear Regression. 

## 3. Results

After the application of the evaluation protocol, the children obtained the following results, which are presented in Table 1 and Table 2.

We can see that the final results are better than the ones obtained in the initial testing. In the case of the Raven test, the initial mean is 39.57 units, which fall into the category of intelligence above the average level. In the final testing, the children registered a mean of 42.58 units, and this implies a transition to a higher category of the I.Q., namely, superior intelligence. We can also see that the value of the standard deviation decreased by one unit, which indicates a more homogeneous distribution of results. We registered an accidental value, where one student solved only nine items in the final testing, although his initial result was normal and close to the collective mean. If he had achieved the same result, then the mean would have been over 43 units, which would be an even better result.

**Figure 1:**
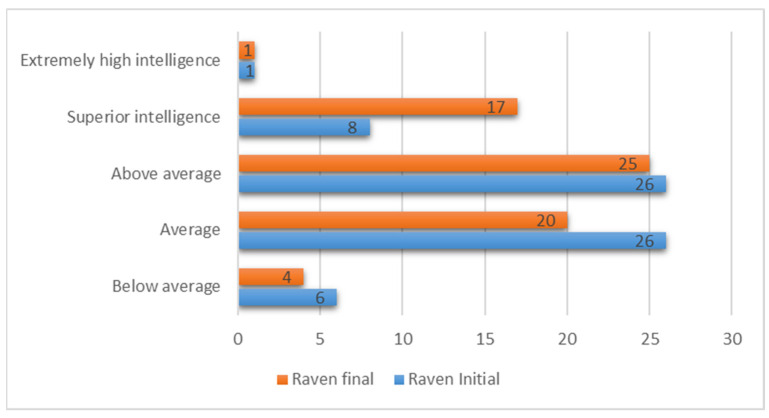
RAVEN results distribution.

In the initial testing, we observed a normal distribution of results (Figure 1) where students with average and above-average intelligence predominated. It is worth noting that a significant percentage (9%) recorded a result below the age average, but even so, at the opposite pole, students with higher intelligence were more numerous (12%). This distribution validates the group of subjects, showing that they form a normal collective of students.

In the case of the final test, we observed an unusual distribution (Figure 1) in which the number of students with superior intelligence was significantly higher than in the initial testing (25%, compared to 12%). Also, as a justification for this change, we saw a 9% reduction in students with average intelligence. It should be noted that the number of students with an intelligence below average decreased, and if we take into account that an accidental score was recorded, the improvement is more hopeful.

At Goodenough testing, the results were normal and the improvement was a collective effort. It can be seen that both the minimum and the maximum values increased and the standard deviation did not change statistically (at least 1 unit), which means the results inside the group kept the same distribution. It is worth mentioning that their final result corresponds to a superior category. 

In order to observe if there was any statistically significant difference between the groups, we used a paired samples *t*-test. As can be seen in Table 2, in every test we registered a value of *p* smaller than 0.05 (0.000, 0.000).

An important aspect of understanding the data obtained was to highlight the percentage of correlation between the individual results of the two tests. Thus, we obtained a strong correlation in both tests (0.769—Raven, 0.693—Goodenough), which indicates that the progress was uniform, extending to the whole group. We can eliminate the variant in which a small number of students registered an exceptional evolution, masking the collective stagnation.

Raven: With a Cohen’s d of 0.39, 65.2% of the final results were above the mean of the initial testing. There was a 60.9% chance that a child picked randomly would have a higher score on the final test.

Goodenough: With a Cohen’s d of 0.60, 72.6% of the final results were above the mean of the initial testing. There was a 66.4% chance that a child picked randomly would have a higher score on the final test.

As we can see in Table 3, multiple R represents the Pearson correlation coefficient value and the 0.41 means that there is a moderate correlation between the registered results. R^2^ shows how much variance the dependent variable can be accounted for by the independent variable. A variance of 0.17 × 100 = 17% in the body schema can be accounted for by the I.Q. coefficient. This means that, on average, the results for body schema drift aside from the regression line by 5.50 units.

Our null hypothesis is that there is no linear relationship between body schema and intelligence. This hypothesis is valid only if the *p* value is >0.05, but in our case, we must reject it and accept the alternative hypothesis that states that there is a linear relationship between the two components that we examine in this article. Because of this result, we can conclude that the linear regression model is significant.

Looking at the intercept value (18.97), we can highlight the most basic version of the equation, which is: Predicted variable = slope × Dependent variable + Intercept. In our case, if we take the 38 units result in the Raven progressive matrices, we can predict that these children should register a value of 31.89 in the Goodenough test, which is above the value corresponding to their age.

As we can see in Table 4, the *p* value is <0.05, which allows us to accept the alternative hypothesis stating that the intercept or slope is not 0, otherwise, the equation of regression would be null.

**Figure 2:**
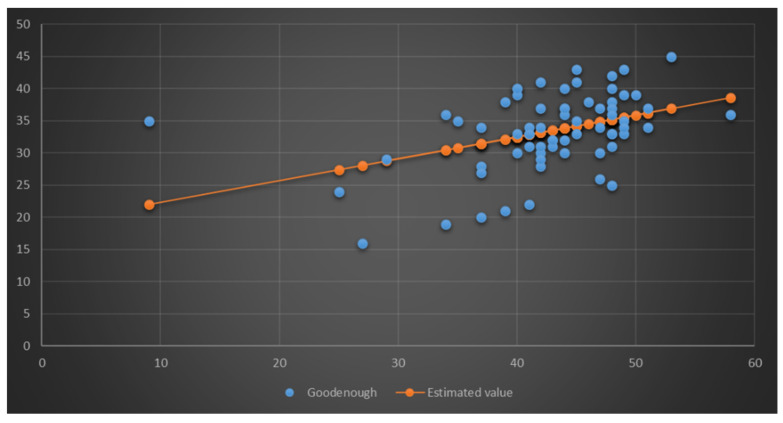
Simple linear regression—Raven–Goodenough.

As we can see in Figure 2, we discovered an ascending regression line between intelligence and body schema. We can observe an accidental result, which influenced to some extent the collective result. When he was asked about his work he simply replied, “I didn’t understand the task”, but during the test, he said that everything was fine and it was actually easy. Because of this, we decided to keep his result as it was.

## 4. Discussion

It is unanimously accepted that the practice of chess develops the level of intelligence, and this hypothesis has been approached by several researchers [21,24,29,54,55]. By its very nature, chess forces the child to work with abstract notions based on solid principles similar to those that govern our lives [56]. The repetitiveness of this exercise aims to develop their own thought filter so that the child will manage, in a more calculated way, the following important decisions in his life.

Since a chess tournament involves a preliminary preparation, during and after it, the time allocated to the analysis of some planned decisions is substantial. The player will have enough information about the newly concluded event to understand the causes of the failure or the key to success. The process of understanding and accepting a reality plays an essential role in how to form the perception of the environment, whether it is the relationship between him and his opponents, or between him and his training colleagues. Understanding and accepting one’s own abilities, but also those of others, triggers a self-awareness at a deep level, thus, the child’s adaptability increases and the transitions from one educational cycle to another are much more easily accepted.

Given these considerations, we wanted to see if the level of intelligence was an index of how the level of the body schema evolves. To provide truthfulness to the results, we chose a test, Raven Progressive Matrices, with a similar solving method to chess. Both involve the reception, analysis, processing and sorting of abstract notions in order to discover the logical principle of correspondence between them. In the case of the body schema, we chose an age-specific testing method that did not involve many restrictive rules. The fact that the student has to draw a man offers freedom of thought that is also found in the game of chess. Although each person has created certain patterns and a cumulative experience, in the game of chess, there are no two identical games, except for the short draws obtained by repeating the moves three times. As in chess, the resources made available to the child can be managed at will. The Goodenough test, by its format, gave us the opportunity to observe several aspects of the evolution of the body schema. The test provides a complete overview, which, in relation to other research focused on the evolution of children, has allowed us to understand how the body schema is formed. The child discovers the body gradually, successively, depending on the extracurricular activities carried out. We found a strong correlation between boys who practice a sport (in our case, football) and the clear, explicit representation of the lower limbs, but the same students completely neglected the shape, place of insertion and proportionality of the arms. There is also a huge difference between the drawings of clothes according to gender. Most of the boys only drew a line that justifies the sleeves of the shirt, while the girls gave fine details of the clothing. The boys neglected simple aspects of the face area, while the girls emphasized these details. Cumulatively, the fine details made the difference, so the veracity of the numerical results was validated.

The Raven progressive matrices highlighted the level of intelligence and the initial results put the children at the border between intelligence above the average level and higher intelligence, and one of the factors correlated with it— also demonstrated in other research—was the practice of chess. The main feature of chess is the need to assimilate a large amount of information and group it into patterns, even if they consist of abstract elements, guided by simple and strict rules, making it an extremely beneficial means of developing the process of thinking.

Extracurricular activities carried out systematically and pursuing a practical purpose educate the child’s character, especially in the context of avoiding distractions. The grading system does not allow the teacher to make a clear ranking, so the child is not affected by a semi-failure in the form of an unsatisfactory grade, because its repair is easy and often comes in the near future. With the transition to the fifth grade, many children face adaptation problems, and the main cause is not their inability to comply with the new requirements, but the deficiencies perpetuated during the primary cycle. It is no coincidence that the results registered at Raven progressive matrices are above average because chess forces the children to think in order to develop their own filter of thought, not forcing them to assimilate raw information that is used immediately. Their long-term memory storage is achieved only in the case of correlations with events that have a strong emotional impact, otherwise, they will disappear after a couple of days. Developing the thought process helps the children to enable a transition from short-term memory to long-term memory, which will help with educational activities. 

We used linear regression to be able to see if the overall Raven test results can be a predictor for those obtained in the Goodenough test. According to the statistical data presented in Table 3, we found that intelligence is a predictor, statistically validated, of the level of the body schema at a percentage of 17%. Although this value does not seem to be high, it should be noted that the formation of the body schema is a continuous process that is influenced by many factors, among which we mention the following: genetics, family environment, social environment, economic environment, education, previous experiences and more. As we can see, the practice of chess can adjust the entourage or small group of friends and the level of education because it is considered non-formal education.

Previous experiences, the genetic factor or the family from which the child comes are variables that we cannot influence through chess, so the percentage obtained is a significant one. The amount of time allocated to practicing chess should also be taken into account because the activity with the greatest impact on self-perception is participation in official tournaments. To be able to compete with real chances, a single hour of chess per week is not enough. Taking this into consideration, we are encouraged by the results discovered.

Given the fact that the attention to detail and the power of concentration influenced the results drastically, it might be a possible explanation for the differences between the initial and final results. It is also noteworthy that even the initial collective score places the group in the first category of the test scale.

In the case of the Goodenough test, the distribution of results is interesting, with the girls having a significantly higher result than the one recorded by the boys. A substantial increase in the collective average can be observed, and this is validated by the test scale. The final collective result increased by about 4 units, and this is the equivalent of moving from one age category to another.

The choice of the test battery was made according to certain arbitrary criteria, and an improvement is necessary in order to be able to generalize the results. Also, duplicating this research for middle school students could provide a better perspective.

We must admit that there are certain research limits, such as the number of participants and the duration of the chess training. We mention that our findings are just a starting point and the conclusions that we have drawn should be verified in further research.

## 5. Conclusions

The level of intelligence is a predictor of the evolution of the body schema. Given that the practice of chess has been correlated with intelligence in numerous studies [21,24,29,54,55], we can say that by practicing chess, the child will benefit from a multilateral development that will increase their integration into various social environments.

Based on our findings, we can assume that intelligence is a predictor, statistically validated, of the level of the body schema at a percentage of 17%. Since many factors influence the evolution of the body schema, such as genetics, family, entourage, education, etc., this percentage is a satisfactory one.

The practice of chess can be considered a non-formal type of education, to ensure a multilateral development of children in primary school.

We also noticed improvements in the level of the body schema, and this can be a key factor in the process of introducing chess into the school curriculum. In addition to the components described above, the practice of chess, through specific competitions, inoculates the idea of fair play and respect that develops the socio-cultural side of the child.

Highlighting the relation between body schema and intelligence represents a starting point for future research.

## Figures and Tables

**Table 1:** Descriptive Statistics.

		N	Min	Max	Mean	Std. Dev.
Raven	Initial	67	13	55	39.57	8.22
	Final		9	58	42.58	7.16
Goodenough	Initial		14	41	29.76	6.25
	Final		16	45	33.45	5.98

**Table 2:** Paired samples *t*-test.

Tests	N	Correlation	t	*p*	Cohen’s d
Raven	67	0.769	−4.587	0.000	0.3867
Goodenough		0.693	−6.262	0.000	0.6005

**Table 3:** Regression statistics—Raven–Goodenough.

Multiple R	R^2^	Adjusted R^2^	Standard Error	Observations
0.41	0.17	0.15	5.50	67

**Table 4:** Simple linear regression—Raven–Goodenough.

	Coefficients	St. Error	t Stat	*p*-Value
Intercept	18.97	4.09	4.64	0.00
X Variable	0.34	0.09	3.59	0.00

## Data Availability

Data can be available for consultation when requested from the corresponding author.
